# Understanding the formation mechanism of consumers’ behavioral intention on Double 11 shopping carnival: Integrating S-O-R and ELM theories

**DOI:** 10.3389/fpsyg.2022.984272

**Published:** 2022-09-14

**Authors:** Wen-Lung Shiau, Mengru Zhou, Chang Liu

**Affiliations:** Department of Business Administration, Zhejiang University of Technology, Hangzhou, China

**Keywords:** behavioral intention, information, ELM, S-O-R, Double 11, experience, involvement

## Abstract

Double 11 shopping carnival, celebrated by the most successful electronic-commerce (e-commerce) Chinese company, Alibaba, has always been the online shopping festival with the highest turnover and involves the largest number of consumers and enterprises in China. This study integrates the elaboration likelihood model (ELM) and stimulus-organism-response (S-O-R) theory to study the dual-processing path of information, which drives customers’ behavioral intention on Double 11. There are 454 valid samples of data are collected, and the research model is tested using the partial least squares method. Results show that in the Double 11 context, two different processing mechanisms affect consumers’ behavioral intention. Thereinto, consumers’ behavioral intention is more affected by the peripheral path than the central path. The affective experience affected by the information stimulus has a greater impact on the behavioral intention than cognitive experience. Furthermore, we find situational involvement have different moderating effects on the relationship between two experiences and behavioral intention.

## Introduction

In recent years, the development of global electronic commerce has grown exponentially, and consumers’ shopping patterns have evolved tremendously. Online shopping has become the first choice of consumers (Rose et al., 2012). In 2020, the share of global online retail in all retail sales increased from 16 to 19%. This implies that the number of innovative e-commerce enterprises has surged, countless traditional enterprises have tried to transform to expand their online business, and millions of consumers are familiar with online shopping. Among them, China’s e-commerce sales are 2.3 trillion USD, accounting for 29% of global e-commerce sales. Concurrently, the huge sale volumes from China’s e-commerce market have promoted the development of the Asia-Pacific region, making it the largest regional e-commerce market worldwide. In China, the Alibaba Group is known as the global e-commerce giant. Double 11 shopping carnival is a precedent for the e-commerce shopping carnival celebrated by the Tmall Business-to-Consumer shopping platform on November 11, 2009. For over 10 years, Double 11 has always been China’s largest online shopping carnival. In 2021, Double 11 transactions reached 540.3 billion CNY (77.9 billion USD), which is equivalent to each Chinese spending 387.35 CNY (60.77 USD). Since 2009, the Gross Merchandise Volume (GMV) of Double 11 has escalated every year. The GMV during Double 11 in 2021 has increased by 42.1 billion CNY over the same period in 2020, at a growth rate of 8.45%.

Faced with the success of Double 11, researchers began to pay attention to Double 11. At the same time, some scholars pointed out that although Double 11 maintained a successful trend, its market share was declining with the emergence of various online shopping festivals, such as Jindong 618 (Chen and Cheung, 2020). Therefore, to maintain the sustainable achievements of Double 11, it is crucial to pay attention to the factors influencing consumers’ purchasing decisions on Double 11. Several scholars focus on the influence of information on consumers’ behavioral intention on Double 11. For example, Xu et al. (2017) unveiled that Double 11 comprises complex and large amounts of information. To improve the quality of the decision-making process, customers need comprehensive information to maximize the value of the decision and minimize decision errors. Yu et al. (2018) believed that Double 11 provides consumers with extensive products and information, allowing them to freely make selections within a specified period. When consumers believe that the information is beneficial to them and provides them with a good experience, they will be motivated to make a purchase. Therefore, customers are influenced by information when making discretionary purchasing decisions.

Although many studies have proven the importance of information (Bhattacherjee, 2001; Shiau et al., 2020), these studies only revealed that information will ultimately change consumer behavior. Few studies are concerned about the actual differences in the formation of behaviors. When consumers make the same behavior, they may adopt different paths to process information because of different situations (Bhattacherjee and Sanford, 2006). To understand this difference elaborately, scholars use the elaboration likelihood model (ELM) theory to illustrate how consumers’ attitudes and behavioral intention can be influenced or altered through two different information elaboration channels after being stimulated by external key factors (Petty et al., 1981; Kang and Namkung, 2018; Yao and Shao, 2021). ELM has a rich empirical research tradition in social psychology and marketing (Petty and Cacioppo, 1986; Lord et al., 1995) and is being applied to research in online shopping research (Wang, 2014; Yang, 2015). In addition, some scholars uncovered that an intermediate mechanism may exist in the path of individual information processing (Cao et al., 2020), which could demonstrate the influence process of information on customers’ attitudes and behaviors but has been ignored by most of the studies applied ELM theory. Double 11 is a special online shopping carnival, which has the most abundant information (Chen and Cheung, 2020). In the context of Double 11, most scholars only focus on the direct influence of information on behavior (Xu et al., 2017; Yang et al., 2018). However, we believe that consumers will influence their behavioral intention through different information processing paths in Double 11. Therefore, this study integrates stimulus-organism-response (S-O-R) and ELM theories to explore how consumers are stimulated by information on Double 11 and how they process information through different paths of internal states such as cognition and affective experience, and in turn affect behavioral intention.

Furthermore, due to the particularity of the Internet as a shopping channel, online shopping completely differs from shopping through traditional channels. The Double 11 differs from ordinary online shopping, given its temporary and special nature. Therefore, we set situational involvement as a moderating variable to analyze the relationship between experience and behavioral intention, which will help further explain the influence on consumer behavioral intentions on Double 11.

To achieve the research goals, the following two research questions are raised:

**RQ1:** What are the two information processing paths that affect consumer behavioral intention?

**RQ2:** How does situational involvement moderate the effects of consumer experience on the intention to participate in Double 11?

Our findings offer some valuable contributions as follows. First, we integrate S-O-R and ELM theories to explore how behavioral intention on Double 11 is affected, which extends the original ELM theory that ignores the internal processing mechanism of information. Second, this research explores the processing mechanism of information through cognitive and affective experiences. The researchers find that familiarity as a central path cue, affects affective experience more than cognitive experience. Contrarily, perceived information credibility, as a peripheral path cue, affects cognitive experience more than affective experience. Furthermore, affective experience plays a greater role than cognitive experience in affecting behavioral intention. Third, investigating the moderating effects of situational involvement on the relationship between experience and intention explains that consumers with different extents of situational involvement have significant differences in the impact of cognitive experience on behavioral intention. Finally, these findings can help Double 11’s platforms and merchants understand the determinants that affect consumers’ purchasing decisions, understand customers’ preferences, further improve the quality of products and services, and eventually gain more market share.

The rest of this paper is organized as follows. “Theoretical background” reviews the related literature on S-O-R and ELM theories. Then, “Research model and hypotheses” proposes the theoretical model and hypotheses. “Research methodology” details the research method. “Results” presents the data analysis process and results. “Discussion” presents the key findings from the results, theoretical and managerial implications, limitations, and future research. “Conclusion” draws the conclusion.

## Theoretical background

### Elaboration likelihood model

ELM has a rich empirical research tradition in social psychology and marketing. Through the central and peripheral paths, it explains how individuals’ behaviors and attitudes change (Petty and Cacioppo, 1986). Specifically, the central path refers to the process through which individuals receive relevant information and earnestly check its comparative advantage. The peripheral route refers to individuals’ judgment of the target behaviors through simple clues or reasoning to change the corresponding attitude and behavior without in-depth thinking (Cao et al., 2020). The central route mainly differs from the peripheral route in two aspects. First, the central route processes the arguments and clues of information, whereas the peripheral route refers to some heuristic clues for an individual to process information. Second, the central route requires careful consideration, which requests the information receiver to invest more cognitive energy. Contrarily, the information processed by the peripheral route must only concentrate on the obvious clues (Petty and Briñol, 2012). Hence, the role of ELM aims to prove that consumers may have distinct processing paths for different information, which will influence their behavior.

The ELM is a framework theory that explains the process of individual information processing without requiring fixed factors in the framework (Tam and Ho, 2005). In various situations, researchers may choose different variables based on the characteristics of the situation, e.g., source credibility (Sui and Zhang, 2021), argument quality (Bhattacherjee and Sanford, 2006), the quality of online consumer reviews (Park et al., 2007), performed product quality and brand awareness (Chen and Cheung, 2020), and reviewers’ creditworthy (Limei and Wei, 2020). Given that consumers’ information sources on Double 11 mostly belong to the Tmall platform, the perceived information credibility is the extent to which one perceives Double 11’s recommendation or review information as believable, true, or factual (Fan and Lederman, 2018), and relevant to their credibility of the Double 11 platform (Xu et al., 2017). The perceived information credibility is the extent to which one perceives Double 11’s recommendation or review information as believable, true, or factual (Fan and Lederman, 2018). Therefore, we choose perceived information credibility as the trigger of the peripheral path, which is consistent with the results of previous studies (Bhattacherjee and Sanford, 2006; Limei and Wei, 2020).

Furthermore, in the context of Double 11, familiarity refers to the consumer’s degree of acquaintance with Double 11, which includes knowledge of the vendor and platform and understanding its relevant procedures, such as searching for products, information on the purchase interface, and how to purchase goods (Kim et al., 2008). From the perspective of information processing, Marks (1981) believed that consumers will compare the present information with the information they have obtained through familiarity and change their behavior based on the benefits of the current situation and the past. Double 11 has been operating for over 10 years, consumers believe they have been highly familiar with the discount level and purchase process of Double 11. Given that Double 11 is innovating every year, such as changes in the reduction and exemption rules, the innovation of interactive games, and the changes in the channels for snatch red envelopes. When customers participate in Double 11 again, the perceived of familiarity will affect their judgment on the information on Double 11. For example, consumers who are extremely familiar with various discounts on Double 11 will compare existing information with past information. Therefore, we think familiarity may trigger the central path. This paper selects the perceived information credibility and familiarity that are highly related to the information as path triggers to explore the different responses of consumers to the stimuli they receive.

### Stimulus-organism-response

S-O-R theory is derived from environmental psychology proposed by Mehrabian and Russell (1974) to explore the factors affecting individual behavior, which suggests all aspects of the environment act as stimuli to jointly affect individuals’ internal state (O) and then drive their behavioral response (R). That is to say, the environmental stimuli, conceptualized as an influence that arouses an individual, do not directly affect individual behavior, but indirectly affect behavior through an internal process of intervention, such as perceptions, feelings, experience, and thoughts (Bagozzi, 1986). For example, Huang (2012) verified consumer behaviors may be indirectly affected by the mediating role of intrinsic virtual experiences.

In the online shopping context, we define stimulus as the sum of all the cues that consumers can see and hear (Eroglu et al., 2001). For consumers, the entire shopping environment is reduced to a computer or mobile phone screen. Consumers’ behavior depends on the information presented on the screen. On Double 11, consumers browse relevant information (such as product reviews, discounts, gifting activities, and game rules) on the Tmall platform. When consumers believe they are familiar with the information or the information they get is credible, their experience will increase, and their purchase intention will be ultimately affected. Thus, we can view familiarity and perceived information credibility as two important trigger stimuli. In addition, within the S-O-R framework, consumers’ responses may follow cognitive or affective internal processing, which stems from the reactions evoked in the organism after exposure to the stimulus. In some previous studies, the organism can be understood from an experiential perspective (Huang, 2012; Kim and Johnson, 2016). Similarly, Frow and Payne (2007) believe that the formation of experience is the consumer’s internal processing of incoming stimuli. Meyer and Schwager (2007) defined offline consumer experience as the internal and subjective reaction of consumers to the direct or indirect contact of the merchant. In the online retailing context, customer’s online experience is a psychologically subjective response to the e-retail environment (Martin et al., 2015). Specifically, cognitional and affective experiences are considered by many scholars as two important dimensions of experience. Cognitive experience refers to connection with thinking or conscious mental processes (Martin et al., 2015). Affective experience involves one’s affective system through the generation of moods, feelings, and emotions. Previous studies have generally summarized ELM theory as a cognitive model. Some scholars have realized this problem and proposed that cognitive and affective experiences are equally important, and the important role of affective experience in information processing should not be ignored, both of which impact behavior (Fishbein and Ajzen, 1975). Therefore, we regard consumers’ perception of the received information, such as the perceived information credibility and familiarity (S), will trigger consumers’ internal affective and cognitional reactions (O), as the dual-process paths, and then evoke a subsequent change in behavior (R). This can explain the specific state of an individual’s information processing and be an extension of the information processing path of ELM theory.

### Involvement

Involvement is defined as an individual’s perceived relevance or importance of a product or service based on inherent needs, values, and interests (Zaichkowsky, 1985). Consumers with a higher degree of involvement examine the relevant social and psychological environment and focus more on the performance and price of the product. Involvement have moderating effects on two information processing paths in ELM, which illustrates that the formation of individuals’ attitudes and behavior are based on their degree of involvement with an issue or a product (Petty et al., 1981; Meng and Choi, 2019). In addition, previous evidence suggests that situational involvement, compared with enduring involvement, is easily affected by the uncertainty related to the purchase situation (Dholakia, 2001). Double 11 is yearly celebrated, at that time, there exist substantial discounting information, interactive games, and festive entertainment activities that are not available in ordinary online shopping activities. Thus, the context of Double 11 is immediate and temporary. When consumers participate in Double 11, if they have a higher involvement, they will check the social and psychological environment surrounding the consumption more carefully and pay more attention to objective factors than the consumers with lower involvement, such as the performance and price (Dholakia, 2001). Therefore, researchers choose situational involvement to moderate the relationship between experiences and behavioral intention.

## Research model and hypotheses

**Figure 1:**
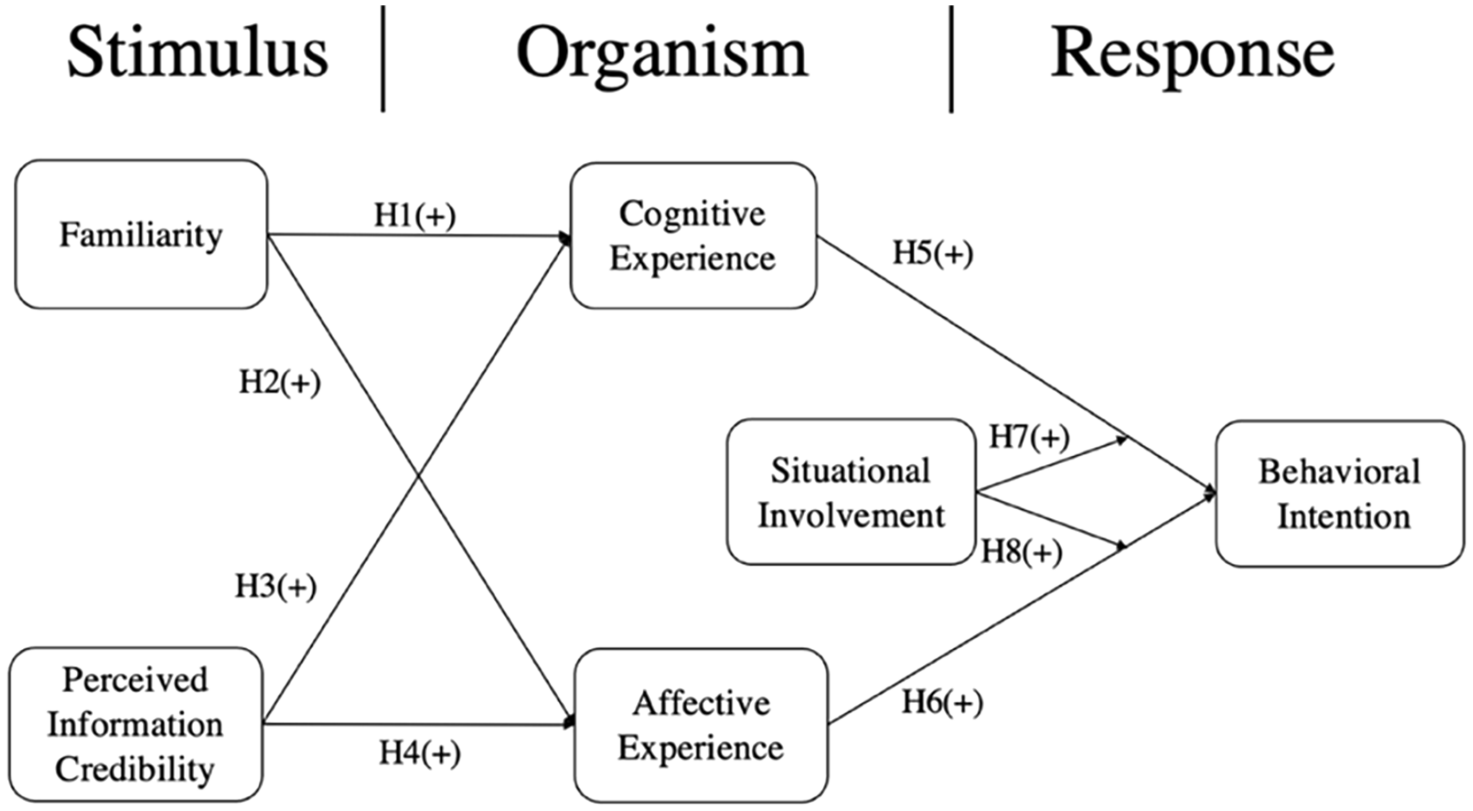
Research model.

In the research model, we integrate S-O-R and ELM theories to be the theoretical framework and adopt familiarity and perceived information credibility as the triggering factors of the two information processing paths. Due to the characteristics of information, familiarity is the central cue, and perceived information credibility is the peripheral cue. Furthermore, cognitive and affective experiences are individual’s internal reactions to information triggering, which may influence the individual’s behavioral intention. Situational involvement is a moderating variable to moderate the relationship between the two consumer experiences and behavioral intention. Figure 1 depicts the proposed conceptual framework.

### Familiarity and consumer experience

In this context, familiarity can positively affect customers’ experience both on the cognitive and affective levels (Bapat, 2017). Fan and Lederman (2018) and Kang et al. (2015) proved the positive effect of familiarity on cognition experience. Moreover, familiarity, which can bring more emotions to consumers, is related to affective experience (Windmann and Kutas, 2001; Kim et al., 2008). Double 11 has been successfully held for 12 consecutive years, with new innovations every year and the growing GMV. Consumers must constantly adapt to new changes and adjust their original knowledge under the new gameplay, which may make them think that they can obtain greater returns in return. Therefore, researchers believe that the more familiar consumers are with Double 11, the stronger their cognitive experience will be. In addition, the familiarity of consumers is gradually increasing, which will make consumers think that they can master the way of participating in Double 11. Therefore, familiarity will produce pleasant and comfortable emotions and even a sense of anticipation. Accordingly, the following hypotheses are proposed:

*H1:* Familiarity positively affects cognitive experience during Double 11 shopping carnival.

*H2:* Familiarity positively affects affective experience during the Double 11 shopping carnival.

### Perceived information credibility and consumer experience

Perceived information credibility represents efficiently distinguishing the useful messages, which is considered the degree to which people believe the recommendation or review information is credible or true. Rose et al. (2011) explained the positive impact of perceived information credibility on experience. Fan and Lederman (2018) suggested that the degree to which consumers believe information is trustworthy is related to the affective experience they acquire in the online shopping context. Moreover, Metzger (2007) conducted an empirical study on how users determine the credibility of network information and pointed out that consumers’ cognitive experience may be influenced by the amount of effort that they will invest in credibility evaluation. During the Double 11, millions of merchants will push extensive information to consumers, which makes the perceived information credibility crucial for consumers to finish the whole purchasing process. When customers perceive that the information provided by Double 11 is credible, they may naturally produce a positive affective experience as well as obtain the economic benefits described in the information and thus gain a good cognitive experience. Hence, the following hypotheses are proposed:

*H3:* Perceived information credibility positively contributes to affective experience during the Double 11 shopping carnival.

*H4:* Perceived information credibility positively contributes to cognitive experience during the Double 11 shopping carnival.

### Consumer experience and behavioral intention

Based on the S-O-R framework, an organism is considered a cognitive or affective intermediary state. It builds up the relationship between outside stimulus and an individual’s response, which is manifested as a specific attitude or behavior change (Conner and Armitage, 1998). Shiau et al. (2021) revealed that cognitive and affective experiences have a positive impact on behavioral intention. In terms of cognition, Lemon and Verhoef (2016) attempted to combine consumer experience with behavior in terms of consumer satisfaction and service quality and found that consumer experience has a positive impact on behavioral intention. In terms of affection, Ning and Hu (2022) confirmed that affective information triggers emotional responses and then influences consumers’ behavioral intention. During the Double 11 period, when consumers have cognitive experience from information, they might participate in Double 11 to buy products even beyond their budget. Concurrently, the affective experience attracts and encourages consumers to participate in the interaction intently and deeply or purchase products. Hence, the following hypotheses are proposed:

*H5:* Cognitive experience significantly and positively affects consumers’ behavioral intention during the Double 11 shopping carnival.

*H6:* Affective experience significantly and positively affects consumers’ behavioral intention during the Double 11 shopping carnival.

### Consumer experience, situational involvement and behavioral intention

Previous studies have confirmed the moderating effect of situational involvement on the relationship between consumer experience and behavioral intention (Mathew and Thomas, 2018). Ma and Atkin (2016) explored that consumer experience affects online shopping intention from two dimensions of cognition and affection, and these relationships will be moderated by situational involvement. Cognitive experience reflects the inner state of consumers changed due to utilitarian reasons, such as the usefulness and practicality of the product or service (Kang et al., 2015; Kim and Johnson, 2016). In the online shopping process, the higher the situational involvement of consumers is, the more their perceptions of utilitarian benefit are (Martín et al., 2011). Furthermore, the level of situational involvement can adjust the influence of consumers’ affective experience on behavioral intention (Martín et al., 2011; Micu et al., 2019). Affective experience reflects consumers’ inner affective state changed through the influence of novel, interesting, and entertaining hedonic services (Bagozzi, 1986; Park and Lee, 2008). The higher the situational involvement of consumers is, the higher their perceptions of hedonic benefits from affective experience (Ha and Lennon, 2010; Hsieh et al., 2014). In Double 11, the higher the situational involvement of consumers, the more sensitive they are to information about the utilitarian benefits that they may obtain. The higher the degree of utilitarian benefits they perceive, the more they are willing to participate in Double 11. Similarly, consumers with higher situational involvement are probably aware that participating in Double 11 will entail a stronger sense of enjoyment, which will lead to more behavioral intention. Hence, the following hypothesis is proposed:

*H7:* The relationship between cognitive experience and customers’ behavioral intention is stronger for the strong situational involvement than for the weak situational involvement during the Double 11 shopping carnival.

*H8:* The relationship between affective experience and customers’ behavioral intention is stronger for the strong situational involvement than for the weak situational involvement during the Double 11 shopping carnival.

## Research methodology

IBM SPSS Statistics 25.0 was used to analyze the samples and explain the descriptive statistics. Furthermore, structural equation modeling is good in the path and factor analysis and especially contributes to presenting the reliability and validity of research results from different perspectives. The significant advantages of Partial Least Squares Structural Equation Modeling (PLS-SEM) include relaxing the assumptions of normal distribution required by the maximum likelihood method used to measure models by Covariance-Based Structural Equation Modeling (CB-SEM). PLS-SEM can easily estimate complicated models with small sample sizes (Shiau and Chau, 2016; Hair et al., 2019; Khan et al., 2019; Shiau et al., 2019). Compared with CB-SEM, PLS-SEM is more suitable for this study in the following situations, where the research goal is exploratory research for theory development; where the analysis is for the perspective of prediction; where the structural model is complex; where the structural model includes no less than one formative constructs; where the sample size is smaller owing to a small population; where distribution is lack of normality; and where research requires latent variable scores for consequent analyses (Gefen et al., 2011; Shiau and Chau, 2016; Hair et al., 2019; Khan et al., 2019; Shiau et al., 2019, 2020). Given that our research model is relatively complex with six constructs and eight hypotheses, PLS-SEM is an appropriate method for this study.

### Measurement development

Table 1 shows the operational definitions and the scale items for these constructs, which are adapted to suit the context of Double 11. Specifically, items measuring familiarity (FAM) are adapted from Kim et al. (2008). The measurement of perceived information credibility (PIC) comes from the scale of Fan and Lederman (2018). Measurements of cognitive experience (CE) and affective experience (AE) are adapted from Molinillo et al. (2020). The measurement of behavioral intention (BI) is developed from Kim et al. (2008). Items measuring situational involvement (SI) are adapted from Hong (2015). All the constructs are measured following a seven-point Likert scale from “strongly disagree = 1” to “strongly agree = 7.”

**Table 1:** Operational definitions and scale items of constructs.

**Constructs**	**Measurement item**	**Source**
Familiarity (FAM) Consumer’s degree of acquaintance with Double 11, which includes knowledge of the vendor and platform and understanding its relevant procedures (Kim et al., 2008).	FAM1. I am familiar with Double 11.	Kim et al., 2008
	FAM2. I am familiar with searching for products on the website during Double 11.	
	FAM3. I am familiar with buying products on the website during Double 11.	
	FAM4. I am familiar with the processes of purchasing products from the website during Double 11.	
Perceived Information Credibility (PIC) The extent to which one perceives Double 11 shopping carnival’s recommendation or review information as believable, true, or factual (Fan and Lederman, 2018).	PIC1. I think Double 11’s information is not credible. (Reverse coded)	Fan and Lederman, 2018
	PIC2. I think Double 11’s information is believable.	
	PIC3. I think Double 11’s information is trustworthy.	
	PIC4. I think Double 11’s message is truthful.	
	PIC5.I think Double 11’s message is reliable.	
Cognitive Experience (CE) The cognitive experience is specified as being “connected with thinking or conscious mental processes” (Martin et al., 2015).	CE1. I have ever felt strongly immersed in Double 11’s application.	Molinillo et al., 2020
	CE2. I felt strongly immersed when using Double 11’s application.	
	CE3. Most times I use Double 11’s application I feel strongly immersed.	
	CE4.Every time I use Double 11’s application I feel strongly immersed.	
Affective Experience (AE) The affective experience involves one’s affective system through the generation of moods, feelings and emotions (Martin et al., 2015).	AE1. Double 11 shopping carnival is very happy.	Molinillo et al., 2020
	AE2. Double 11 shopping carnival is very content.	
	AE3. Double 11 shopping carnival is very pleased.	
	AE4. Double 11 shopping carnival is very excited.	
	AE5. Double 11 shopping carnival is very stimulated.	
Situational Involvement (SI) Situational involvement was defined as the consumer’s temporary perception of festival importance that accompanies Double 11’s particular purchase situation based on the consumer’s desire to meet extrinsic goals deriving from the purchase and/or usage of Double 11 (Hong, 2015).	SI1. Double 11 shopping carnival is important.	Hong, 2015
	SI2. Double 11 shopping carnival is excited.	
	SI3. Double 11 shopping carnival is interesting.	
	SI4. Double 11 shopping carnival means a lot.	
	SI5. Double 11 shopping carnival is involving.	
	SI6. Double 11 shopping carnival is needed.	
Behavioral Intention (BI) The likelihood that a consumer actually buys online during Double 11 (Lee et al., 2011).	BI1. I am likely to purchase the products(s) on Double 11.	Kim et al., 2008; Lee et al., 2011
	BI2. I did not intend to purchase the products(s) on Double 11 this year. (Reverse coded)	
	BI3. I am likely to recommend Double 11 to my friends.	
	BI4. I am likely to make another purchase from Double 11 if I need the products that I will buy.	

Familiarity (FAM); Perceived Information Credibility (PIC); Cognitive Experience (CE); Affective Experience (AE); Behavioral Intention (BI); Situational Involvement (SI).

### Participants

Before data collection, a pilot test was conducted with 10 consumers who ever participated in Double 11. They were invited to evaluate the format, structure, length, and semantics of the questionnaire. Afterward, the questionnaire was revised based on their feedback. In addition, before the official questionnaire was issued, 50 questionnaires for the pretest were used to examine the reliability and validity of the scale to ensure the validity of the content. The reliability test results show that the Cronbach’s alpha values of all constructs are between 0.781 and 0.979, which are all higher than 0.7. The composite reliabilities (CR) ranged from 0.856 to 0.984, all exceeding 0.7, which indicates that the questionnaire in this study has high reliability (Bagozzi and Yi, 1988). Given that the factor loading of the measurement item PIC1 is less than 0.5 (Wixom et al., 2001), the item PIC1 was deleted. The average variance extracted (AVE) of each construct all exceeds 0.5 (Chin et al., 2003), illustrating that the questionnaires in this study have satisfactory convergent validity. After ensuring the reliability and validity of the pretest, the formal questionnaires were distributed. Data samples were collected from the largest online survey website Sojump^1^ in China. Compared with traditional paper surveys, online surveys have some advantages in fast response time, cost-efficiency, and cross geographical boundaries (Bhattacherjee, 2001). The main survey was distributed online in 2021, and we received 623 responses. After collecting the sample data, we screened for invalid questionnaires. First, invalid samples include incomplete questionnaires due to missing items and invalid questionnaires due to identical answers. For example, select “7 totally agree” for all questions. These questionnaires may reflect the interviewees did not fill out the questionnaire carefully, so they were deleted. Second, the questionnaire was designed with reverse coded (PIC1 and BI2). If there was a contradiction between positive and negative questions, the questionnaire was deemed invalid. Finally, we deleted sample data that present the respondents who have never participated in “Double 11 Online Shopping Carnival” and thus are not our test subjects. After removing invalid questionnaires, 454 valid questionnaires were finally obtained.

## Results

### Demographic profiles

Table 2 shows the demographics of 454 respondents, of which 64.1% are women and 35.9% are men. The proportions of respondents in the three age groups between 20 and 25, 26 and 30, and 31 and 35 are, respectively, 48.46, 16.30, and 17.84%. This means that most of the respondents are between 21 and 35 years old (82.60%), which is roughly consistent with the age distribution of all participants of Double 11. In addition, over 55% of the respondents have participated in Double 11 for more than 4 years (which means over 4 times), hence most of the respondents are familiar with Double 11.

**Table 2:** Descriptive statistics.

**Demographic variable**	**Classification**	**Number**	**Percentage**
Gender	Male	163	35.90%
	Female	291	64.10%
Age	<20	17	3.74%
	21–25	220	48.46%
	26–30	74	16.30%
	31–35	81	17.84%
	36–40	31	6.83%
	41–45	25	5.51%
	46–50	3	0.66%
	>51	3	0.66%
Education	Junior college or below	82	18.06%
	Bachelor’s degree	243	53.52%
	Master’s degree	121	26.65%
	Doctor’s degree	8	1.76%
How long has it been since your first participation in Double 11?	1 to 3 years	187	41.19%
	4 to 6 years	226	49.78%
	7 years or more	41	9.03%
What is the total shopping expenditure during Double 11? (In CNY)	<200	20	4.41%
	200–499	74	16.30%
	500–999	188	41.41%
	1,000–2,999	138	30.40%
	>3,000	34	7.49%
What is the total number of items purchased during Double 11? (In unit)	1–5	143	31.50%
	6–10	201	44.27%
	11–20	88	19.38%
	>20	22	4.85%
What is the total time spent during Double 11? (In minute)	0–30	73	16.08%
	30–60	212	46.70%
	60–120	105	23.13%
	>120	64	14.10%

### Non-response bias and common method bias

By comparing the gender and age of the early and late respondents, we check whether a problem of non-response bias exists. This method, proposed by Armstrong and Overton (1977), depicts that the late respondents are more likely to be non-respondents than the early respondents. In this study, the first 50 samples of respondents are selected as the earlier respondents, and the last 50 samples are selected as the later respondents. The chi-square test of early and late respondents shows that no significant difference exists in gender and age (*p* > 0.05). Therefore, we rule out the possibility of non-response bias in this study. In addition, common method bias may affect the validity of the results, especially when the data are collected from a single source at a certain time (Podsakoff et al., 2003). In this study, program control methods are used to reduce the degree of common method bias and statistical methods are used for testing. In terms of program control, control methods bias, such as anonymity of the fill-in person and setting up reverse questions are adopted. In statistical testing, we use two methods of statistics for measurement, Harman’s single-factor method and the marker variable method. Harman’s single-factor method can evaluate the degree of bias of the common method, which is to combine items for factor analysis. The combined four factors account for 64.489% of the total variance. The largest factor accounts for 49.460% (the variances explain ranged from 4.511 to 49.460%), and no general factor accounts for more than 50% of the variance. In addition, the results of data analysis using the marker variable method to test common method bias (Chin et al., 2012) show that the marker variable does not significantly affect cognitive experience, affective experience, and behavioral intention. Therefore, in this study, the common method bias is not a serious issue.

### Measurement model

The results in Table 3 show that the factor loadings of all items range from 0.733 to 0.897, all exceeding 0.5 (Wixom et al., 2001). Composite reliability measures the internal consistency of the model, ranging from 0.885 to 0.946, all exceeding 0.7 (Bagozzi and Yi, 1988). All Cronbach’s alpha values fall between 0.775 and 0.928. The average variance extracted (AVE) of each construct ranges from 0.597 to 0.777, all exceeding 0.5 (Chin et al., 2003), demonstrating satisfactory convergent validity. The standardized root mean square residual (SRMR) is 0.048, under the recommended threshold of 0.08, indicating that the model has a good fit for the sample data (Henseler et al., 2015). To evaluate the discriminant validity, both the Fornell–Larcker and the Heterotrait–Monotrait (HTMT) ratio of correlation were examined. As shown in Table 4, the square root of the AVE of each construct is greater than the absolute value of the correlation coefficient between the corresponding constructs (Fornell and Larcker, 1981). Therefore, the results confirmed the model’s discriminant validity. Henseler et al. (2015) proposed the Heterotrait–Monotrait (HTMT) ratio of the correlations and suggested 0.90 as a threshold value for structural models with constructs. In this research, the values ranged from 0.651 to 0.889, which indicated that discriminant validity was established for all constructs of the model, as shown in Table 5. Therefore, the results confirmed the model’s discriminant validity.

**Table 3:** Reliability and validity or measurement model.

**Constructs**	**Item**	**FL**	**Mean**	**SD**	**CA**	$\rho_{A}$	**CR**	**AVE**
Familiarity	FAM1	0.785	5.493	1.218	0.827	0.827	0.885	0.659
	FAM2	0.828	5.654	1.182				
	FAM3	0.801	5.650	1.157				
	FAM4	0.831	5.648	1.149				
Perceived information credibility	PIC1	0.845	5.623	1.148	0.867	0.868	0.909	0.714
	PIC2	0.854	5.412	1.080				
	PIC3	0.823	5.370	1.094				
	PIC4	0.857	5.456	1.065				
Cognitive experience	CE1	0.865	5.196	1.320	0.874	0.875	0.913	0.725
	CE2	0.851	5.339	1.314				
	CE3	0.834	5.172	1.251				
	CE4	0.855	5.187	1.330				
Affective experience	AE1	0.874	5.396	1.396	0.928	0.929	0.946	0.777
	AE2	0.861	5.326	1.384				
	AE3	0.887	5.390	1.317				
	AE4	0.887	5.275	1.339				
	AE5	0.897	5.368	1.412				
Situational involvement	SI1	0.796	5.385	1.317	0.885	0.886	0.913	0.635
	SI2	0.833	5.427	1.179				
	SI3	0.785	5.463	1.166				
	SI4	0.775	5.412	1.213				
	SI5	0.817	5.478	1.214				
	SI6	0.775	5.586	1.105				
Behavioral intention	BI1	0.773	5.930	0.973	0.775	0.781	0.856	0.597
	BI2	0.733	5.974	1.241				
	BI3	0.831	5.513	1.070				
	BI4	0.752	5.674	1.092				

FAM: Familiarity; PIC: Perceived Information Credibility; CE: Cognitive Experience; AE: Affective Experience; BI: Behavioral Intention; SI: Situational Involvement; SD: Standard Deviation; FL: Factor loading; AVE: Average Variance Extracted; CR: Composite Reliability; CA: Cronbach’s Alpha.

**Table 4:** Discriminant validity: Fornell–Larcker Criterion.

**Constructs**	**AE**	**BI**	**CE**	**FAM**	**PIC**	**SI**
AE	0.881					
BI	0.704	0.773				
CE	0.649	0.608	0.852			
FAM	0.632	0.609	0.554	0.811		
PIC	0.664	0.726	0.636	0.586	0.845	
SI	0.700	0.734	0.684	0.725	0.716	0.797

FAM: Familiarity; PIC: Perceived Information Credibility; CE: Cognitive Experience; AE: Affective Experience; BI: Behavioral Intention; SI: Situational Involvement.

**Table 5:** Discriminant validity: Heterotrait–Monotrait Ratio (HTMT).

**Constructs**	**AE**	**BI**	**CE**	**FAM**	**PIC**	**SI**
AE						
BI	0.829					
CE	0.720	0.737				
FAM	0.721	0.756	0.651			
PIC	0.738	0.889	0.727	0.690		
SI	0.772	0.882	0.777	0.848	0.815	

FAM: Familiarity; PIC: Perceived Information Credibility; CE: Cognitive Experience; AE: Affective Experience; BI: Behavioral Intention; SI: Situational Involvement.

### Structural model

**Figure 2:**
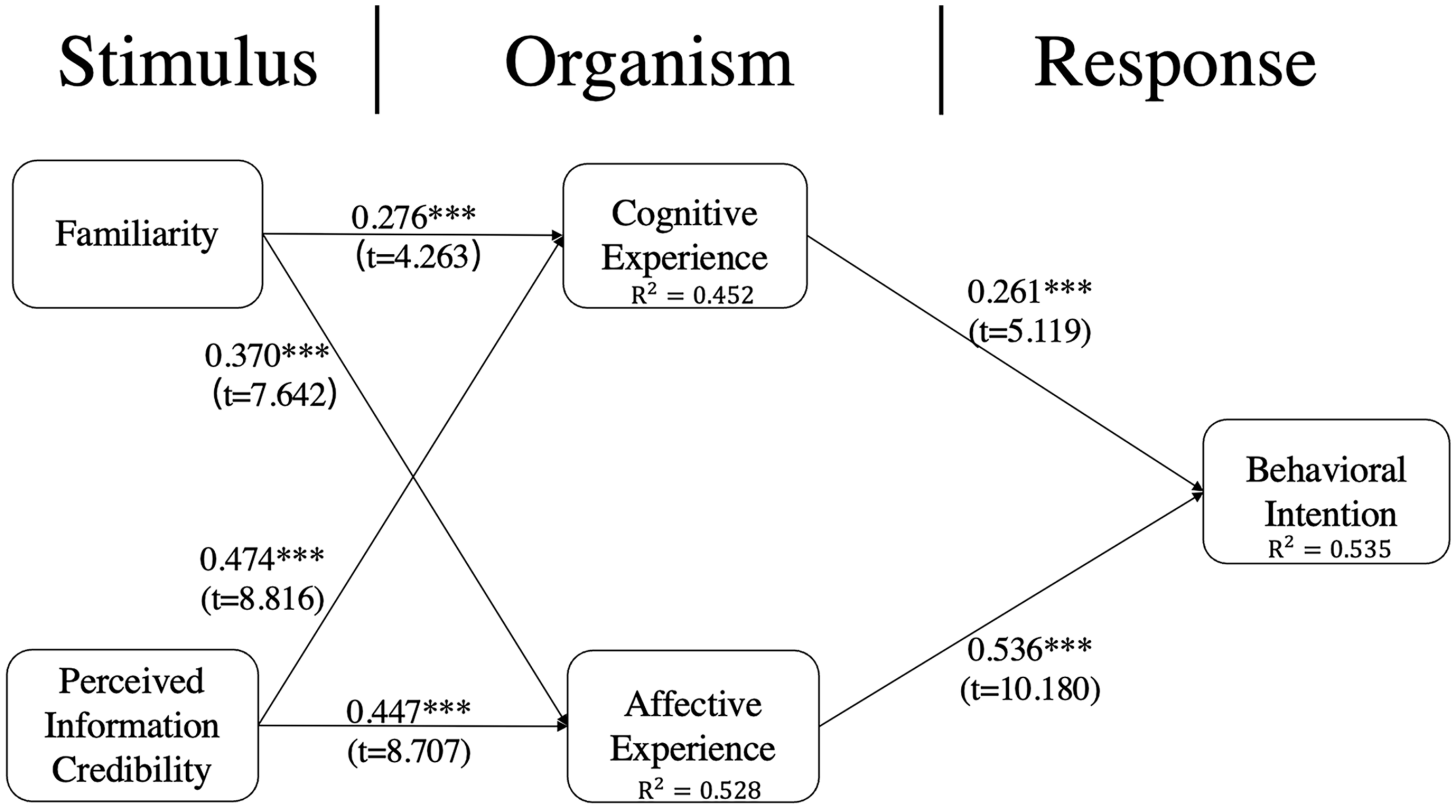
Research model Results of the structure model. ^*^p < 0.05; ^**^p < 0.01; ^***^p < 0.001.

The results of the structural model and path coefficients are shown in Figure 2. The Bootstrap resampling method (5,000 times) is used to ascertain the significance of the structural model paths. Familiarity significantly and positively affects cognitive experience (*β* = 0.276, *t* = 4.263, *p* < 0.001) and affective experience (*β* = 0.370, *t* = 7.642, *p* < 0.001). The perceived information credibility also significantly and positively affects cognitive experience (*β* = 0.474, *t* = 8.816, *p* < 0.001) and affective experience (*β* = 0.447, *t* = 8.707, *p* < 0.001). This study also reveals the positive relationship between consumer experience and behavioral intention. Cognitive experience significantly affects behavioral intention (*β* = 0.261, *t* = 5.119, *p* < 0.001), and affective experience significantly affects behavioral intention (*β* = 0.536, *t* = 10.180, *p* < 0.001). Furthermore, we calculate the explained variance (R^2^) of each endogenous construct (Hair et al., 2014). The model explains 45.2% of the variance for cognitive experience, 52.8% of the variance for affective experience, and 53.5% of the variance for behavioral intention. Moreover, we examined collinearity. The variance inflation factor (VIF) values of the constructs are below the recommended (Neter and Wassermann, 1974). Specifically, the outer VIF values range from 1.417 to 3.083, and the inner VIF values are all no more than 3. All results show collinearity is not a critical issue.

### Moderation effect analysis

We use a standardized method to create moderator. This method can effectively avoid the collinearity problem (Marsh et al., 2007; Hayes, 2013). The two-stage approach to deal with the moderator was adopted by us. To present more clearly how situational involvement affects the relationship between consumer experience and behavioral intention more clearly, we show Model 1 without the moderating effect and Model 2 with the moderating effect. Model 1 only includes the main model variables, such as familiarity, perceived information credibility, cognitive experience, affective experience, and behavioral intention. Situational involvement as a moderating variable and interaction terms obtained by multiplying the moderating variable and independent variables are added to Model 2. The results of the moderating effects are shown in Table 6. From Models 1 to 2, R^2^ increases from 0.535 to 0.629, verifying the importance of situational involvement to behavioral intention. The results show that situational involvement significantly and positively moderates the relationship between cognitive experience and behavioral intention (Situational Involvement × Cognitive Experience: *β* = 0.099, *t* = 2.076, *p* < 0.05, *f*^2^ = 0.022), but has a non-significant moderating effect on the relationship between affective experience and behavioral intention (Situational Involvement × Affective Experience: *β* = −0.008, *t* = 0.147, *p* > 0.05, *f^2^* = 0.000).

**Table 6:** Regression results for moderation effects.

**Variable**	**Model 1**			**Model 2**		
	**CE**	**AE**	**INT**	**CE**	**AE**	**INT**
FAM	0.276^***^ (4.263)	0.370^***^ (7.642)		0.276^***^ (4.160)	0.370^***^ (7.690)	
PIC	0.474^***^ (8.816)	0.447^***^ (8.707)		0.473^***^ (8.728)	0.468^***^ (8.703)	
CE			0.261^***^ (5.119)			0.105^*^ (2.200)
AE			0.536^***^ (10.180)			0.335^***^ (6.582)
CE^*^SI						0.099^*^ (2.076)
AE^*^SI						−0.008 (0.147)
$R^{2}$	0.452	0.528	0.535	0.454	0.530	0.629

Familiarity (FAM); Perceived Information Credibility (PIC); Cognitive Experience (CE); Affective Experience (AE); Behavioral Intention (BI); Situational Involvement (SI).

* *p* < 0.05;

*** *p* < 0.001.

**Figure 3:**
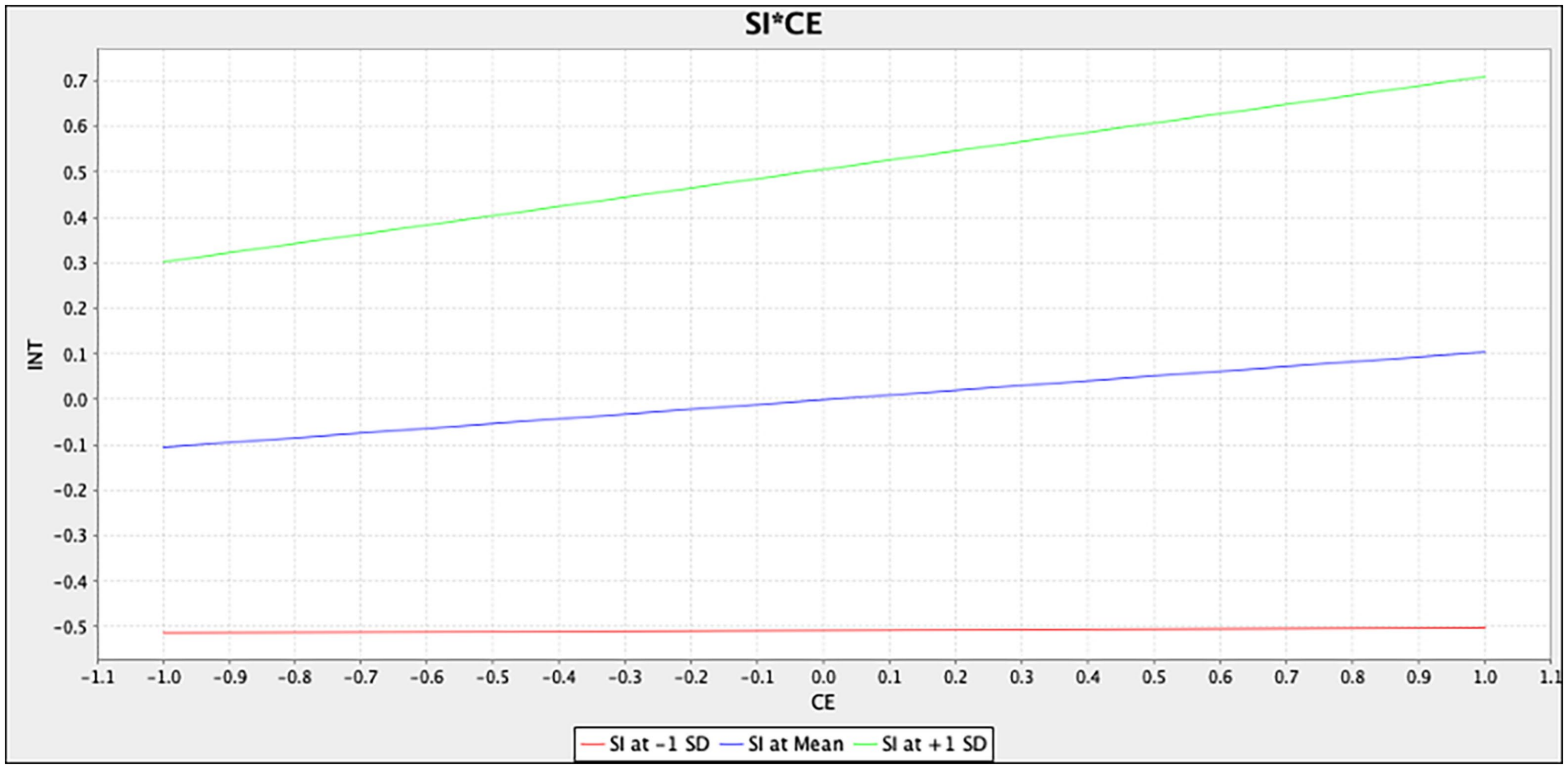
Moderating effect of situational involvement on the relationship cognitive experience and behavioral intention.

**Figure 4:**
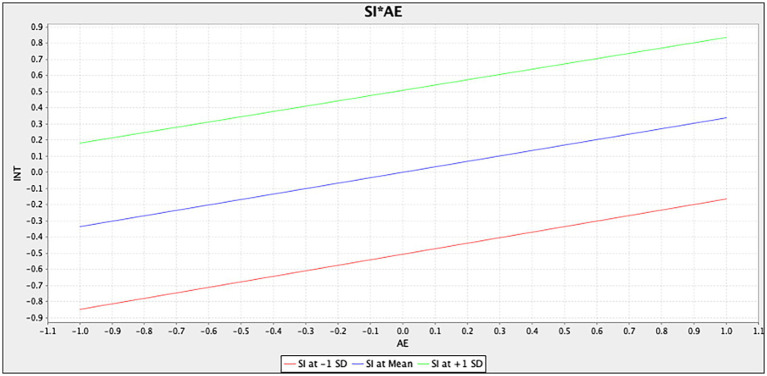
Moderating effect of situational involvement on the relationship affective experience and behavioral intention.

Furthermore, Figure 3 shows the simple slope analysis of situational involvement moderating the relationship between cognitive experience and behavioral intention. The simple slope diagram of situational involvement shows that the relationship between cognitive experience and behavioral intention increases more quickly in customers with high situational involvement [situational involvement at +1 standard deviation (SD)] than in customers with low situational involvement (situational involvement at −1 SD). This finding indicates situational involvement has a significantly positive moderating effect on cognitive experience and behavioral intention. Correspondingly, Figure 4 shows the simple slope analysis of situational involvement moderating the relationship between affective experience and behavioral intention. The simple slope diagram of situational involvement shows that the relationship between affective experience and behavioral intention increases similarly in customers with high situational involvement (situational involvement at +1 SD) compared with customers with low situational involvement (situational involvement at −1 SD). Situational involvement has a non-significant moderating effect on the association between affective experience and behavioral intention.

## Discussion

Among the trigger factors of the information processing routes, the results show that familiarity as a central cue has a significant positive impact on cognitive and affective experiences, which is consistent with the research results of Bapat (2017) and Kim et al. (2008), thus supporting H1 and H2. This means that consumer familiarity enables consumers to obtain cognitive experience while affecting their affective response and producing pleasant emotions. The more consumers are familiar with Double 11, the more interested they are in exploring the details of relevant information and in investing effort in the platform (Iglesias et al., 2011). The more detailed the process of exploration, the more invisible discounts they will find and the stronger the cognitive experience they will obtain. Therefore, consumer familiarity with Double 11 is related to their cognitive experience and affects their purchasing intention (Rahman and Mannan, 2018). Similarly, consumers who are familiar with Double 11 understand the rules of the platform better and may be more easily immersed because they can fully participate and experience happiness, thus obtaining more affective experience.

The results reveal that the perceived information credibility has significant positive impacts on cognitive and affective experiences, thus supporting H3 and H4, which is consistent with previous research results (Pae et al., 2002; Lee et al., 2011; Wang et al., 2017). Consumers’ judgment on the credibility of information on Double 11 is evaluated in a careful and serious process, including the appropriateness, relevance, sufficiency, and authority of information (Wathen and Burkell, 2002). The higher degree of the credibility of the Double 11 information perceived by consumers, the deeper the cognitive evaluation will be, and the stronger cognitive experience they will obtain. In the context of Double 11, once customers trust the message conveyed by information, they will believe they can experience the most cost-effective shopping moment of the year. In addition, if consumers believe that the information on Double 11 is highly credible, they will be happy and satisfied when they wait for the opportunity to buy at the bottom price and imagine they will own their favorite products. It suggests that high perceived information credibility will provide consumers with a positive affective experience.

Additionally, this study shows that consumers’ cognitive and affective experiences positively impact behavioral intention, thus supporting H5 and H6. In the Double 11 context, experience plays a key role in consumer behavior, which is consistent with previous studies (Khalifa and Liu, 2007; Rose et al., 2011; Molinillo et al., 2020). This illustrates that those consumers experiencing the economic benefits brought by Double 11 have some cognitive experience. This kind of good utilitarian experience will encourage consumers to participate in Double 11. In addition to economic value factors, aesthetic and emotional factors in the online shopping environment may lead to consumers’ purchase intention (Martin et al., 2015). Merchants on Double 11 often satisfy consumers’ affective needs by providing good interactive interfaces, convenient purchase methods, intimate customer services, and a lively festive atmosphere. The affective experience that consumers obtain from these services will increase their intention (Bloch et al., 1989). Therefore, cognitive and affective experiences are key factors in forming consumer behavioral intention during Double 11.

Furthermore, situational involvement has a significant positive moderated effect on the relationship between cognitional experience and behavioral intention, thus supporting H7. The reason is because that consumers with higher situational involvement will pay more attention to whether Double 11 meets their expectations based on their inherent needs, values, and interests. The cognitive experience of customers with higher situational involvement represents to have a higher level of perceived utilitarian benefits for various discount information, such as special offers, gifts, and promotions. The greater utilitarian incentives they perceive on Double 11 influence consumers to make more purchases. However, interestingly, no significant moderating effect on the relationship exists between affective experience and behavioral intention, thus rejecting H8. This is inconsistent with previous research results (Meng and Choi, 2019; Sharma et al., 2019). The reason may be if consumers have a pleasant and happy affective experience, these enthusiastic emotions will reduce the consumers’ investment in thinking and accelerate the organisms’ reaction process and trigger the unplanned, instant decision-making purchase behavior (Hopwood, 1991). Therefore, regardless of the level of consumer situational involvement, the generation of behavioral intention is not significantly influenced by the perception of hedonic benefits under pleasurable affective experience.

Table 7 presents some interesting findings. First, surprisingly, this study reveals that the peripheral path of processing information generated by perceived information credibility as an information stimulus has a greater impact on intention, with a total effect of 0.200. Contrastingly, the central path of processing information generated by familiarity as the information stimulus has a lower impact on behavioral intention, with a total effect of 0.153. This illustrates that consumers familiar with Double 11 who compare their previous experience with the new Double 11’s optimal purchase plan or new interactive gameplay have less influence on their behavioral intention than consumers who directly believe that Double 11’s offer information is credible. This shows that, in the face of Double 11 shopping carnival where the gameplay is updated every year, people’s purchase behavior will be more affected by the credibility of information rather than information familiarity, and people’s behavioral intention is often more dependent on the fast path of non-in-depth thinking.

**Table 7:** Effects of factors.

**Factors**	**Effects**	**Cognitive experience**	**Affective experience**	**Behavioral intention**
Familiarity	Direct effects	0.276	0.370	—
	Indirect effects	—	—	0.153
	Total effects	0.276	0.370	0.153
Perceived information credibility	Direct effects	0.474	0.447	—
	Indirect effects	—	—	0.200
	Total effects	0.474	0.447	0.200
Cognitive experience	Direct effects			0.105
	Indirect effects			—
	Total effects			0.105
Affective experience	Direct effects			0.335
	Indirect effects			—
	Total effects			0.335

Second, we found that in the processing part of the organism, perceived information credibility produces more cognitive experience, whereas familiarity produces more affective experience. This represents that when customers believe that the information is credible, they will obtain a greater utilitarian experience. For example, they may think they will get the highest benefit of the whole year as stated in the promotional information, and if they miss this high time for purchasing, they will miss the lowest price. Contrastingly, the familiarity generated by people frequently participating in the Double 11 will produce more affective experience rather than cognitive experience. For example, thinking of the joyful emotions generated by participating in different interactive games every year, receiving red envelopes and the carnival shopping atmosphere will create a positive sense of expectation.

Third, in the relationship between organism and response, affective experience has a much greater effect on behavioral intention than cognitive experience. When Double 11 is widely recognized as the shopping festival with the greatest benefits from discounts, it is affective experience that primarily affects people’s participating intention. Although this sounds incredible, it seems to be in line with the actual situation. The reason why Double 11 does not simply call itself a shopping festival but a shopping carnival, shows that the functions realized in its own positioning are not only because it is merely rational shopping, but shopping in a carnival atmosphere. The carnival is related to emotions of delight, such as playful, enjoyable, and happiness (Xu et al., 2017). The carnival mood will greatly influence shopping intention. This also corresponds to the so-called “Hands-chopping people” phenomenon of passionate consumption and explains the high return rate caused by regret after over-enthusiastic consumption.

### Theoretical implications

This study has several theoretical implications. First, we integrate S-O-R and ELM theories to explore when people receive information, how their internal states are affected by such information stimuli, and how they process the information in the dual-process paths, which subsequently drive their behavioral responses. Different from the previous studies on influence processes, most scholars who applied the ELM theory only explored the distinction between the two routes but ignored the internal reaction of individuals in the information processing. That is, the information used as clues might go through specific processing routes. Through the theoretical perspective of S-O-R theory, we address experience as a necessary internal reaction for individuals to process information *via* the ELM dual-process path, which expands the depth of interpretation of the formation of the dual paths of ELM theory.

Second, based on the characteristics of the experience, the mechanisms are divided into two dimensions in this study, cognitive experience, and affective experience. The central path and the peripheral path are, respectively, processed by cognitive experience and affective experience. The interesting finding is that the central path that requires in-depth thinking more affects the affective experience, on the contrary, the peripheral path that less effortful processing of information more affects the cognitive experience. This not only highlights the information richness of the context of Double 11 but also proves the complexity of dealing with Double 11 information. Furthermore, in terms of the influence of experience on the behavioral intention of Double 11, affective experience plays a greater role than cognitive experience when considering the two information processing pathways integrally. This discovery breaks the inherent evaluation of ELM theory that previous studies tend to generally summarize the ELM theory as a cognitive model. This model expands the judgment and application of ELM theory.

Third, compared to the choice of enduring involvement as the moderating variable in the common studies applied ELM theory, we adopt situational involvement as a temporary perception of the importance of Double 11. We find that the cognitive experience of consumers with higher situational involvement has a greater impact on behavioral intentions, while the impact of affective experience on behavioral intentions is not significantly different from that of consumers with lower situational involvement. Furthermore, this research applies the ELM theory to the field of shopping carnivals. Previous studies have applied the ELM theory to online shopping, but it is rarely applied to some specific online shopping contexts.

### Practical implications

This study focuses on the driving effect of the dual processing path of information on consumers’ behavioral intention, proving the importance of information to the Double 11. When customers receive certain information, they will process information in different ways to form consumer experience, and finally, exert decisive influences on their behavior. Therefore, enterprises and merchants need to constantly pay attention to the consumer experience, and constantly develop products or services that can improve online shopping experience, which is an important direction for enterprises and businesses to continuously improve and innovate. To be specific, this research indicated that familiarity and perceived information credibility are information cues affecting behavioral intentions through customer experience. Therefore, improving levels of familiarity and perceived information credibility will encourage consumers to participate in Double 11. Concurrently, the platform should formulate corresponding policies to enhance the online consumer experience.

First, the online consumer experience can be improved by increasing the perceived information credibility of consumers, thereby enhancing behavioral intention. From the cognitive perspective, merchants should add information that consumers care about, such as un-beautified real images of products and product price trends to enhance consumers’ awareness of product authenticity and prices. From the affective perspective, merchants should make full use of entertaining interactive methods and expand the festive atmosphere of Double 11 to improve consumers’ affective experience. Merchants should make full use of entertainment-oriented interaction to establish consumer trust and enhance consumer affective experience with more reliable information. For example, game rewards can be timelier and more varied. The platform should establish a perfect reward system and give different levels of gifts to consumers according to their participation, ranging from red envelopes to limited prizes (Tmall anniversary handwork and celebrity Double 11 signature cards), so that participants can get corresponding prizes if they participate in Double 11. This approach will increase consumers’ sense of authenticity and trust, thus increasing their affective experience.

Second, merchants and platforms should pay more attention to consumer familiarity. First, consumers’ familiarity with festival can be enhanced to improve their cognitive experience. When consumers are familiar with the Double 11 rules, they can understand how to get the maximum benefits of full discount reduction, thus increasing their cognitive experience. Therefore, merchants must maintain the core rules so that consumers will find it easy to deal with information on the next Double 11. In addition, merchants should also pay more attention to how to consider the affective experience of increasing familiarity for consumers. For example, planning team of Double 11 can add more rewards to the game or invite more celebrities to participate in the interactive game and explain the rules of the game. These methods can attract consumers to spend more time to familiar.

Consumer experience is closely related to their behavioral intention. As a bridge between merchants and consumers, the platform side not only provides services to both parties but also formulates rules to restrict both parties to conduct fair and equal transactions. A considerable source of customer experience is the basic service standards and benefit guarantee mechanism provided by the platform. Therefore, the improvement of platform services is of great help to enhance consumer cognitive and affective experiences. The platform should optimize the content of the service and enhance communication with consumer to bring them pleasure. For example, developing a special interactive interface and setting discounts for Double 11. In addition, various kinds of shopping carnival have emerged. To improve the competitiveness of Double 11, the platform can conduct a questionnaire survey about consumer experience to understand the needs of consumers. The planning team of Double 11 can adjust the presentation of product information through the questionnaire results to bring better experience to consumers.

Finally, this study shows that situational involvement can significantly affect the relationship between cognitive experience and behavioral intention. Consumers with high situational involvement will be concerned about the benefits they can get from participating in Double 11. The more utilitarian motivation consumers have, they are more likely to buy more goods. Platforms and merchants can increase the classification of consumers and set different rules for different groups. For example, rank consumers based on browsing time, set basic and simple rules for users who browse less, and add hidden rewards for users who browse more.

### Limitations and future work

The limitations of this study provide additional insights for future research. First, given that participants (enterprises and consumers) of Double 11 have spread to over 200 countries, future research can expand the scope of research worldwide. Second, with the increase of online shopping carnivals, further studies are warranted to extend these findings to other shopping carnivals to help enterprises increase sales. Third, the extended ELM framework theory, with high explanatory power, should be more widely used to analyze the formation of user behavioral intentions in information systems and services.

## Conclusion

As global e-commerce has entered a period of rapid expansion, numerous companies and e-commerce platforms have sent various profit-inducing messages to snatch consumers. Consumers must screen the online shopping information of the Internet “information explosion,” and the process of information processing will lead to their final behavioral intention. The Tmall Double 11 online shopping carnival, of which GMV has grown continuously for more than 12 years, has always led the e-commerce industry’s promotions. Therefore, the influence of consumers’ information processing paths on behavioral intention on Double 11 must be explored. This research expands ELM by integrating S-O-R theory, takes cognitive and affective experiences as the necessary links of information processing, and analyzes how a dual-path information process based on familiarity and perceived information credibility affects customers’ behavioral intention. We have obtained 454 valid sample data and analyzed them through the PLS-SEM. Our results show that consumers’ intention to participate in Double 11 is affected by the peripheral path greater than the central path. In the central path, information stimulus influences affective experience more, whereas in the peripheral path, information stimulus influences cognitive experience more. Furthermore, in the internal reaction process of the individual, it is the affective experience that has a relatively large impact on the behavioral intention. In addition, situational involvement has a significant positive moderating effect on the relationship between cognitive experience and behavioral intention. Our model explains 45.4% of the affective experience, 53.0% of the affective experience, and 62.9% of the variance in behavioral intention.

Through the integration of S-O-R and ELM theories, this study provides an in-depth discussion on the process of consumers receiving information from the Double 11, which can be used as an analytical method to study the individual behavioral decisions of online shopping carnival. Furthermore, the verification of the moderating effect of situational involvement highlights the differences in the relationship between cognitive experience and behavioral intention of consumers with high and low situational involvement. It reveals that the difference in the information processing path of consumers with different types of perception or motivation drive different changes in behavioral intention. Based on the results of this study, an exciting possibility is the application of the expanded ELM framework theory combined with the S-O-R theory not only to the Double 11 shopping carnival but also to other studies that might be more valuable for enhancing specific forms of knowledge.

## Data availability statement

The raw data supporting the conclusions of this article will be made available by the authors, without undue reservation.

## Author contributions

All authors listed have made a substantial, direct, and intellectual contribution to the work, and approved it for publication.

## Funding

This research is supported by Major Project of National Social Science Foundation of China (19ZDA078) on the Mechanism and Path about Technological Standard and Intellectual Property Synergistically Promoting Digital Industry. Also, this research is supported by the 2021 Zhejiang University of Technology Humanities and Social Sciences Pre-research Fund Project (grant number SKY-ZX-20210179).

## Conflict of interest

The authors declare that the research was conducted in the absence of any commercial or financial relationships that could be construed as a potential conflict of interest.

## Publisher’s note

All claims expressed in this article are solely those of the authors and do not necessarily represent those of their affiliated organizations, or those of the publisher, the editors and the reviewers. Any product that may be evaluated in this article, or claim that may be made by its manufacturer, is not guaranteed or endorsed by the publisher.

## Footnotes

^1^ www.wjx.com
